# The Development of an Evidence-Based App to Predict the Need for Enhanced Care for People with Dementia

**DOI:** 10.1192/bjo.2025.10174

**Published:** 2025-06-20

**Authors:** Sabina London, Shanquan Chen, Emad Sidhom, Christoph Mueller, Benjamin Underwood

**Affiliations:** 1 University of Cambridge, Cambridge, United Kingdom; 2 University of Connecticut School of Medicine, Farmington, USA; 3 London School of Hygiene & Tropical Medicine, London, United Kingdom; 4 King’s College London, London, United Kingdom

## Abstract

**Aims:** Dementia is a pressing global health challenge affecting more than 50 million people worldwide with a net impact on the global economy of 1 trillion USD. One aspect of the condition is the potential for deterioration to the point where a crisis situation occurs requiring emergency intense psychiatric support, either in the form of intense community care or admission to an inpatient facility. Such care is only needed for a minority of patients. If patients can be identified at the point of diagnosis, it raises the potential for stratified care pathways for those at highest risk with the aim of improving outcomes. Our previous work from two United Kingdom sites found that younger, male patients, and those with impaired cognition were at risk of deteriorating. In this study, we aimed to create mathematical models of risk and use the results to develop a mobile application that is ready for clinical use.

**Methods:** Using on our retrospective cohort study (*n*=253,260) we have identified several epidemiological, and behavioural factors that showed the highest association with subsequent need for enhanced care. The top 10 variables were selected using dominance analysis and systematic regression in Cambridgeshire and the models externally validated in a second data set from London. The same threshold was used for classifying patients into the high and low risk groups as in our previous retrospective study.

**Results:** The top predictive variables in Cambridgeshire were age (OR: 0.97, p<0.001), gender (OR: 1.74, p<0.001), marital status (OR: 1.34, p<0.05), dementia subtype (OR<0.61, p<0.05), and the following 6 Health of the Nation Outcome Score (HoNOS) subcategories: behaviour (OR: 1.43, p<0.001), hallucinations (OR: 1.20, p<0.05), cognitive (OR: 1.19, p<0.05), disability (OR: 0.79, p<0.001), other mental and behavioural problems (OR: 1.23, p<0.01) and relationships (OR: 1.26, p<0.01). The AUC was 0.74–0.79 for 1–4 years after diagnosis in Cambridgeshire with a similar AUC of 0.74 in the validation dataset in London. The resulting PREDICDEM app is a tool which can be used to stratify people in terms of risk at the point of dementia diagnosis.

**Conclusion:** We have used routinely collected clinical data to model risk of needing enhanced care in dementia. This data can be incorporated into models of risk prediction and into a smartphone app. This will allow stratification of patients at the time of diagnosis and facilitate trials of interventions to decrease the risk of crisis events.

